# Supplementary figures and images for: Epigenetic suppression of SLFN11 in germinal center B-cells during B-cell development
Source: PLoS One. 2021 Jan 29;16(1):e0237554. doi: 10.1371/journal.pone.0237554 (PMC7846023; doi:10.1371/journal.pone.0237554)

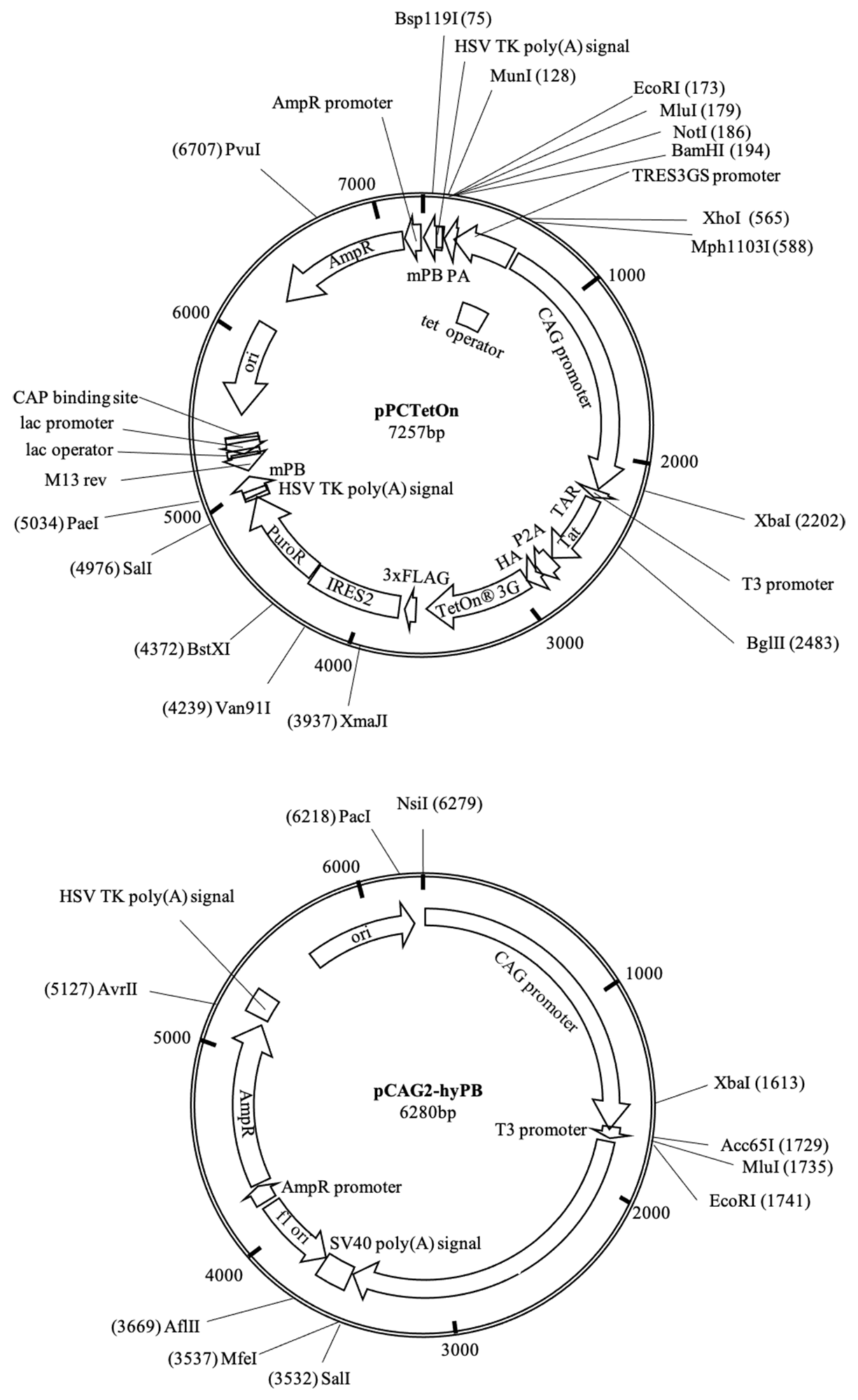

Supplement: S1 Fig — (TIF) [file pone.0237554.s001.tif]

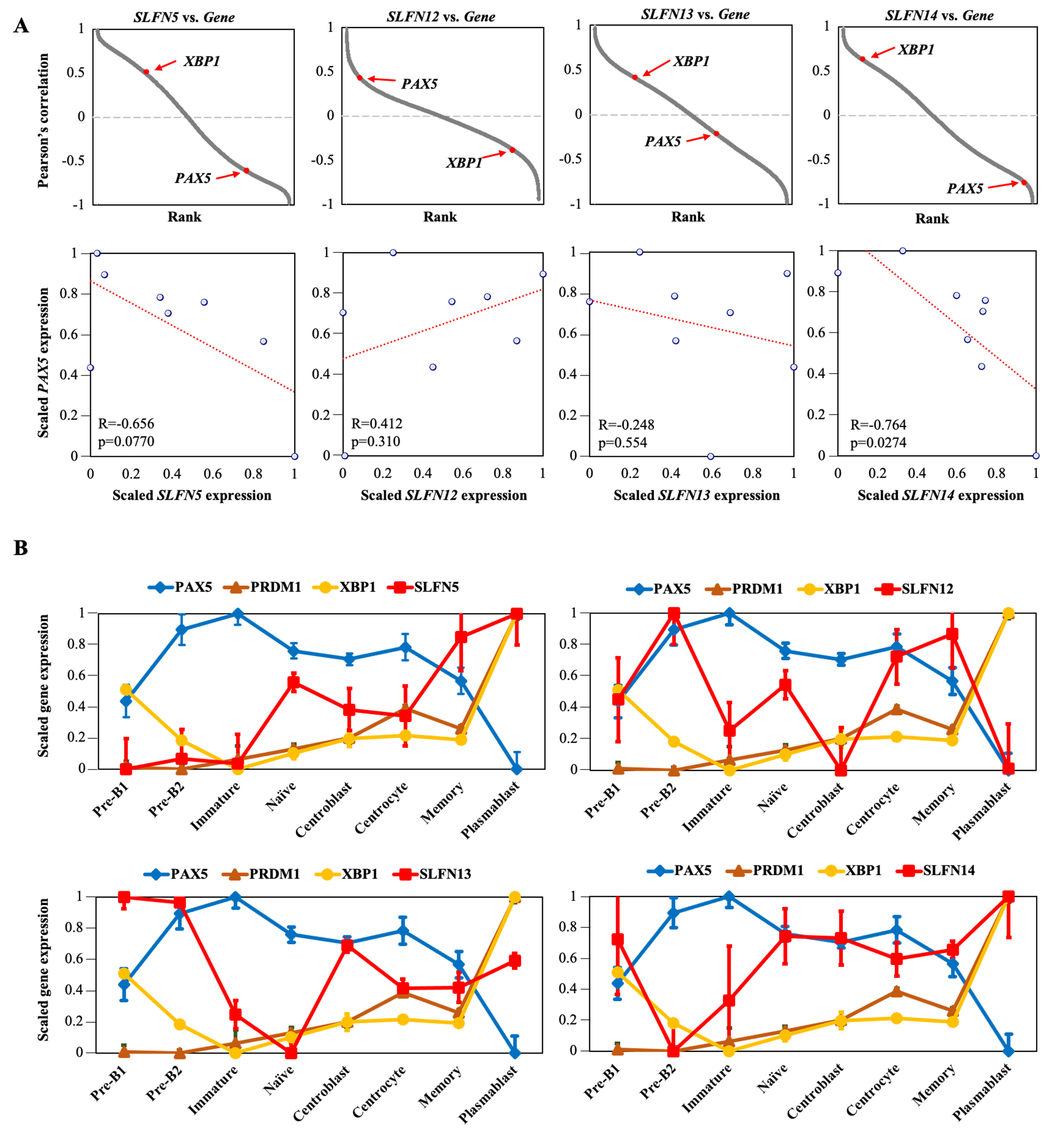

Supplement: S2 Fig — (A) Upper: Pearson’s correlation between SLFN family members (SLFN5, SLFN12, SLFN13, SLFN14) and all the other genes. The genes are ordered from the highest correlation (left) to the lowest correlation (right). Lower: microarray gene expression plot of SLFN family members and PAX5. Precursor (Pre)-B1 cells, precursor (Pre)-B2 and immature B-cells are were taken from human bone marrow (n = 5), and naïve B-cells, centroblasts, centrocytes, memory B-cells and plasmablasts were taken from human tonsil (n = 6). Pearson’s correlation (R), P-value (p) and regression line (red dotted line) are shown. (B) Microarray gene expression profile (log2) of selected genes (PAX5, PRDM1, XBP1, SLFN family members) in human B-cells from bone marrow and tonsil. Dots correspond to group means ± SE. (TIF) [file pone.0237554.s002.tif]

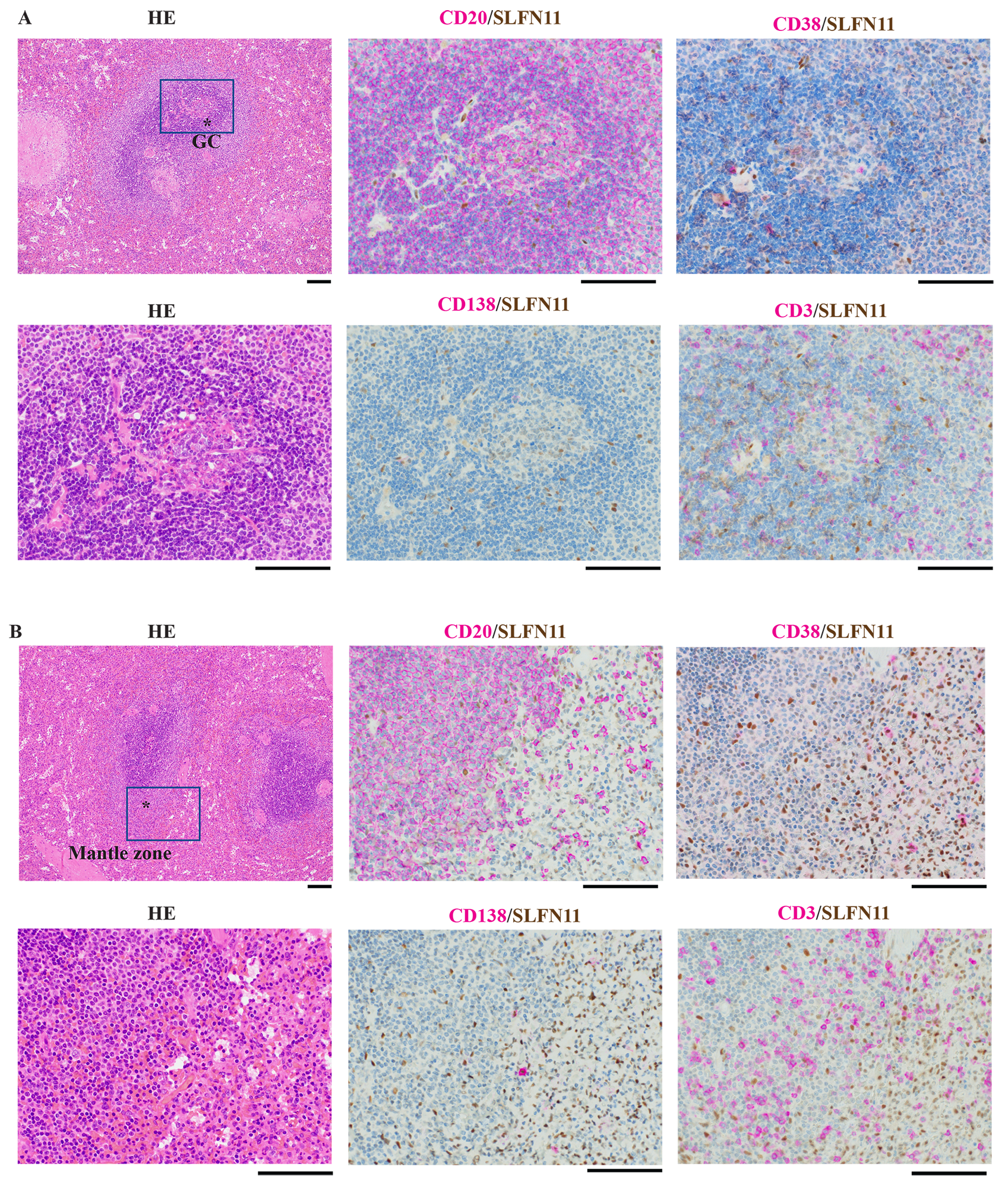

Supplement: S3 Fig — Immunohistochemical staining of human spleen tissue. The samples were stained with hematoxylin eosin (HE). For dual staining, SLFN11 was stained with DAB (brown) and CD markers (CD3, CD20, CD38 and CD138) were with HRP (purple). Original magnification: x10 and x40. Scale bars are 100 μm. GC: germinal center. (A) Germinal center in spleen tissue. (B) Mantle zone and cortex in spleen tissue. (TIF) [file pone.0237554.s003.tif]

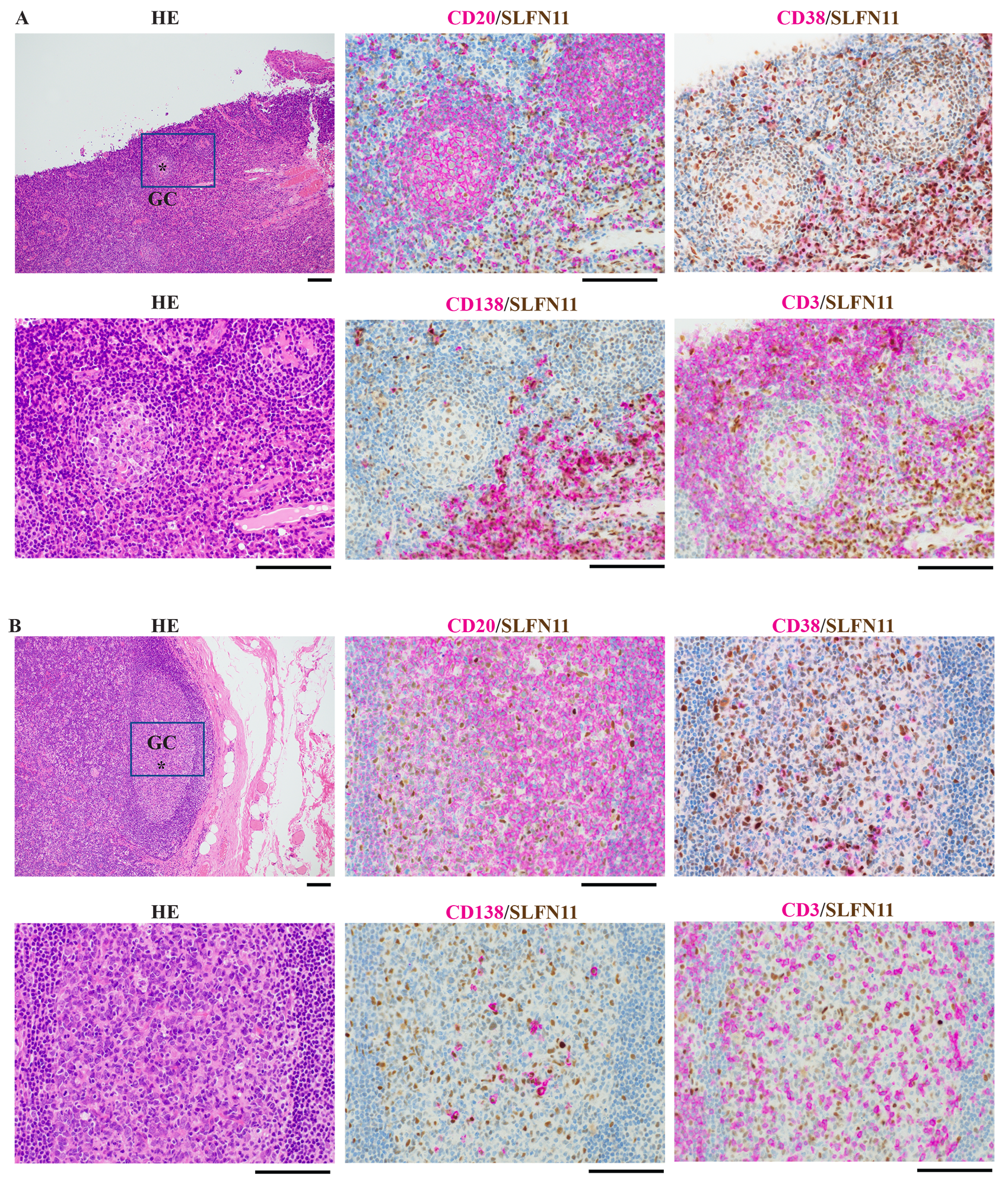

Supplement: S4 Fig — Immunohistochemical staining of human tonsil and lymph node tissue. The samples were stained with hematoxylin eosin (HE). For dual staining, SLFN11 was stained with DAB (brown) and CD markers (CD3, CD20, CD38 and CD138) were with HRP (purple). Original magnification: x10 and x40. Scale bars are 100 μm. GC: germinal center. (A) Germinal center, mantle zone and cortex in tonsil tissue. (B) Germinal center in lymph node tissue. (TIF) [file pone.0237554.s004.tif]

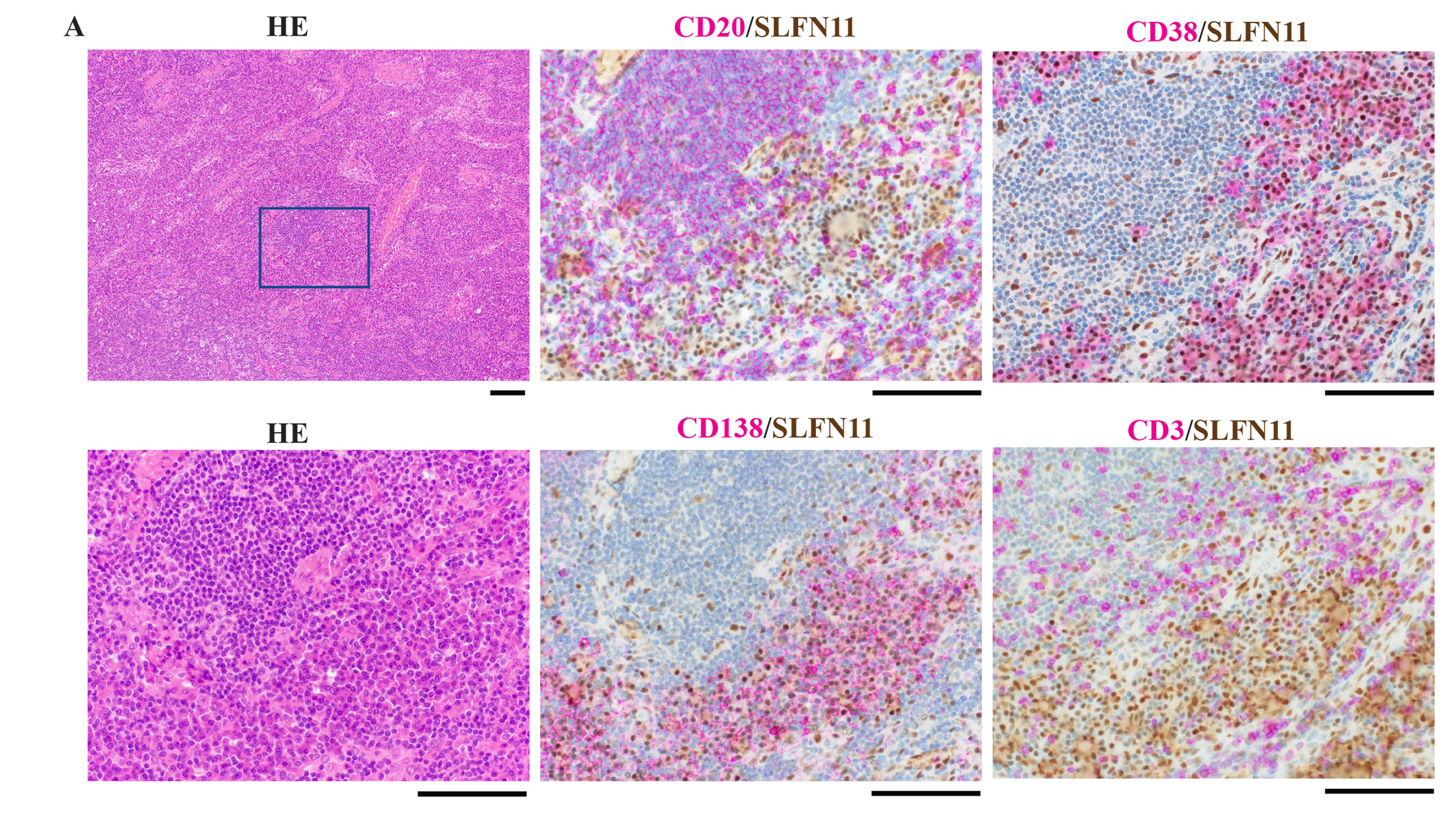

Supplement: S5 Fig — Immunohistochemical staining of human lymph node tissue. The samples were stained with hematoxylin eosin (HE). For dual staining, SLFN11 was stained with DAB (brown) and CD markers (CD3, CD20, CD38 and CD138) were with HRP (purple). Original magnification: x10 and x40. Scale bars are 100 μm. (A) Non-germinal center region in lymph node tissue. (TIF) [file pone.0237554.s005.tif]
