# Supplementary material for: Epigenetic suppression of SLFN11 in germinal center B-cells during B-cell development
Source: PLoS One. 2021 Jan 29;16(1):e0237554. doi: 10.1371/journal.pone.0237554 (PMC7846023; doi:10.1371/journal.pone.0237554)

Point-by-point answers to the reviewers

The list of changes and answers are indicated below (in blue) with the comments of the reviewers included in *italic* before our answers to facilitate the review process.

Reviewers' comments to the Author

Reviewer #1: *SLFN11, a putative DNA/RNA helicase, has been implicated in DNA damage response to platinum-derivatives, topoisomerase inhibitors, PARP inhibitors and replication inhibitors. SLFN11 expression has been shown regulated by epigenetic modifications of DNA and histones in different cell types and tissues. In this paper, Fumiya Moribe et. al. investigated the regulatory mechanisms of SLFN11 expression and its potential roles in B cells by mining publicly available microarray gene expression data and experimentally tested the relationship between SLFN11 expression using IHC and cell viability. They found that SLFN11 expression is epigenetically regulated during B-cell differentiation, and it is typically suppressed in germinal center B cells(GCBs). Moreover, epigenetic activation of SLFN11 in lymphomas of GCB origin enhanced the susceptibility of lymphoma cells to a DNA-damaging agent.*

Overall the experimental design is logical and the data analysis method is sound. A major weakness is that not all the data are supportive of their conclusions and some results appear to be preliminary and not validated experimentally. There are many grammar and spelling errors; the manuscript should be proofread by a native English speaker. In addition, the manuscript should be carefully revised to clarify the rationales and the results need to be interpreted properly.

(Answer) Thank you very much for your positive comments and constructive suggestions. In the revised manuscript, we added new data to consolidate our conclusions. They are included in the revised Figure 2 & Figures S2-S4 (dual immunohistochemical staining for SLFN11 and CD20/CD38/CD138/CD3), Figure 4A (DNA methylation plot for *SLFN11* promoter) and Figure 5A (drug activity data for AraC across 39 B-cell-derived cancers). We have also carefully checked and edited our manuscript to clarify the rationale of our experiments and the interpretation of the results. Moreover, the revised manuscript has been proofread by a native English speaker. Thank you.

The specific comments are listed below:

1. *Line 182-183, the results do not support this conclusion;*

(Answer) Thank you for your suggestions. In the revised manuscript, we write “Thus, among *SLFN*s, *SLFN11* uniquely showed parallel expression profile compared to *PRDM1* and *XBP1* and reverse expression profile with respect to *PAX5* across B-cell development.” instead of the original sentence “Thus, *SLFN11* but no other *SLFNs* expression can be controlled during B-cell development under the same regulatory system for *PRDM1* and *XBP1*.”

2. Figure 2: It needs to clarify how many samples were used in the IHC experiments. A statistical analysis should be performed with clearly labeled sample numbers. In addition, SLFN11 is also not expressed in B cells in Non-GCB regions. How SLFN11 expression is regulated in B cells in non-GCB regions should be explained.

(Answer) Thank you very much for your suggestions. As suggested, in the revised manuscript, we increased the number of samples as well as B-cell markers to consolidate our conclusions. We employed six samples from three

lymphatic tissue types (including each two lymph nodes, tonsils and spleen samples). In addition to the dual staining of CD20 (a marker of premature B cells) and SLFN11, we performed dual staining of CD38 and CD138 (markers of differentiated B-cells) with SLFN11 to determine SLFN11 expression in plasmablasts and plasmacytes. Overall, the results revealed that SLFN11 is downregulated in GCBs (CD20-positive cells in GCs) and is upregulated in plasmablasts (CD38-positive in GCs) and plasmacytes (CD38-positive and CD138-positive in cortex). We added these results in Figure 2A-C as shown below and Figure S2-4 in the revised manuscript. Moreover, we scored the SLFN11-positive population (%) to statistically support our findings by visual inspection. While only 0.8% of CD20-positive cells were SLFN11-positive, 89% and 77% of CD38-positive and CD138-positive cells, respectively, were SLFN11-positive (Figure 2D below, **$p<0.0001$).

Additionally, to distinguish T-cells from B-cells, we performed dual staining for CD3 (a marker of T cells) and SLFN11. We found that 0.5% of CD3-positive cells were SLFN11-positive (Fig2B-D below).

As for the upregulation of SLFN11 in non-GCB regions, we could not clarify the precise mechanism of the upregulation in this study. However, our data (Figs 3-5) imply that histone modification could be key for the regulation of SLFN11 in B-cells. This point has been included in our revised manuscript.

(Figure 2)

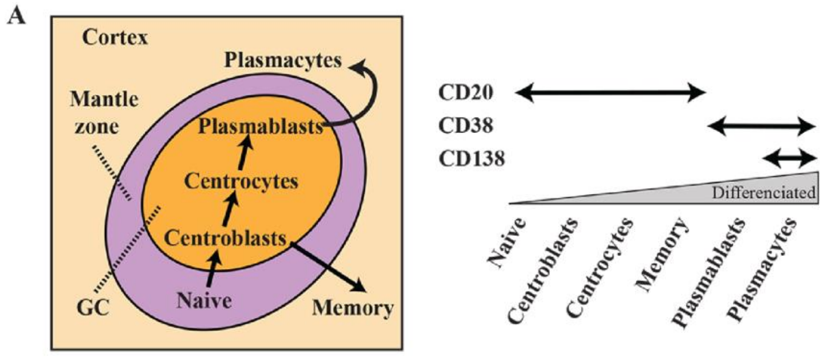

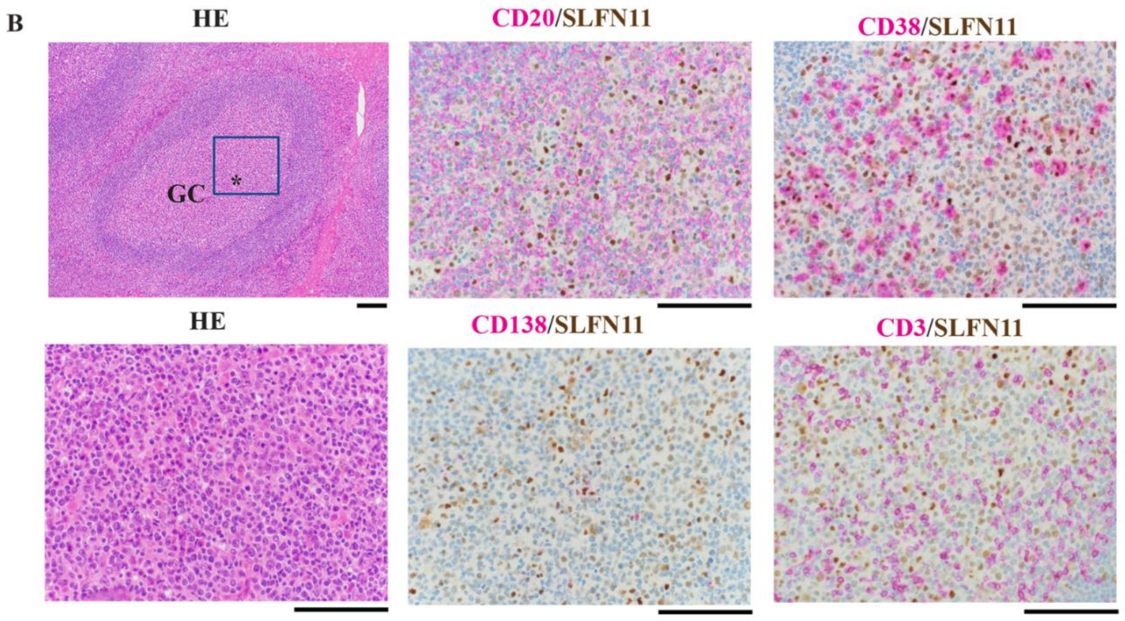

C

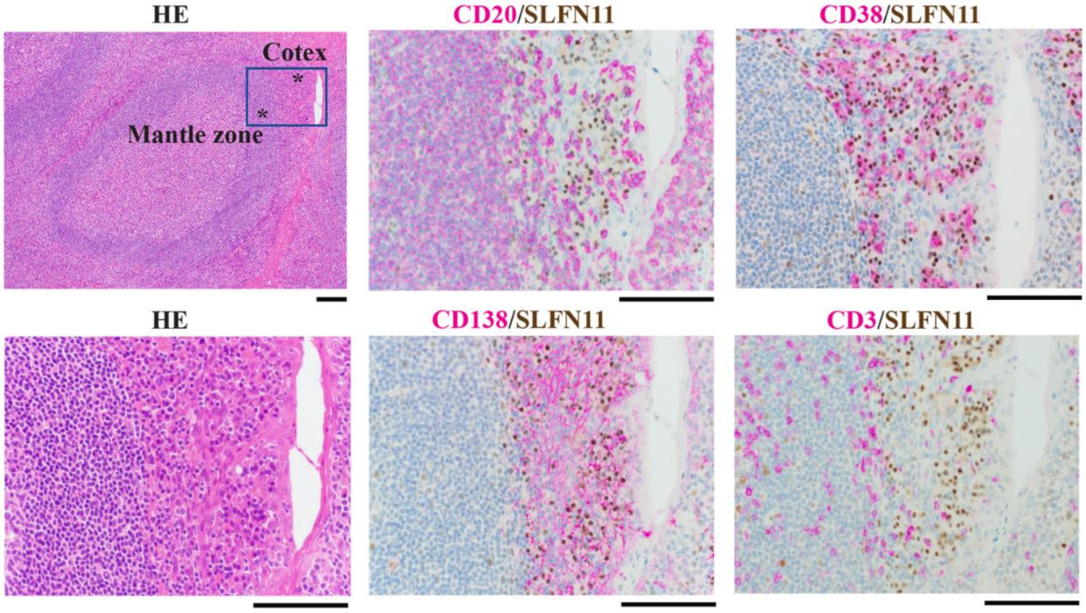

D

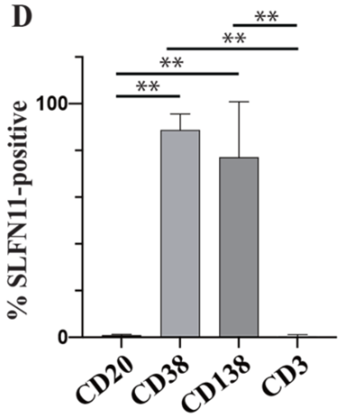

3. The dynamic expression of SLFN11 during B cell development should be validated in both mouse or human samples using IHC.

(Answer) Thank you. To validate the dynamic expression change of SLFN11 during B-cell development, we performed dual IHC with several markers for B-cells (Fig 2). Please see our answers to point # 2 above. Importantly, validation in mouse samples is currently not feasible as SLFN11 ortholog has not been identified among the different SLFN11 murine genes.

4. Line 305-307, the results do not reconcile with this conclusion. To draw the conclusion, the authors should examine whether SLFN11 depletion can sensitize the cells to cytosine arabinoside(AraC).

(Answer) Thank you for your suggestions. To answer your question, we worked hard to make SLFN11 KO cells in SU-DHL6 and other GCB cell lines. However, SU-DHL6 did not express SLFN11 high enough to be detected by WB under the treatments of epigenetic modifiers, and we were unable to validate candidate clones of SLFN11-KO. For follicular lymphoma cell lines, FL18, FL218 and FL318 did not form single colonies after the transfection of SLFN11-KO vectors. For these reasons, we have been unable to establish SLFN11-KO cell lines in the GCB cells used in this study. Alternatively, to examine the relationship between *SLFN11* expression and sensitivity to AraC in

GCB cells, we mined publicly available data on *SLFN11* expression and drug activity of AraC. *SLFN11* expression was found significantly correlated to the activity of AraC in B-cell-derived cancer cell lines. These data have been included in the revised Figure 5A (below). Moreover, we previously reported that the synergistic effect by the combination of HDACi and a DNA damaging agent (camptothecin) is SLFN11-dependent (i.e., the synergy was not observed in SLFN11-KO cells) (Tang SW, et al. Clin Cancer Res. 2018. [26]). Based on this, we conclude that activation of SLFN11 expression by epigenetic modifiers can enhance the activity of AraC. Nevertheless, since we failed to generate KO cells in GCB cells, we rephrased the original sentence and stated, "These results indicate that the combination of the epigenetic modifiers that enhance SLFN11 expression can improve the response in GCB lymphoma cells to AraC" in the revised manuscript.

(Figure 5A)

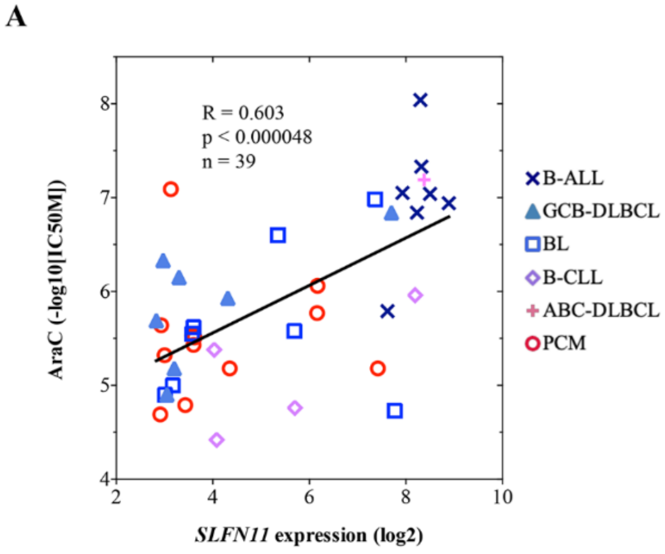

5. Line 355-356, the results do not support this conclusion.

(Answer) An increased number of studies indicate that SLFN11 is a plausible target to sensitize tumor cells to cytotoxic chemotherapy (Coussy F, et al. Sci Transl Med. 2020 [17], Gardner EE, et al. Cancer Cell. 2017 [24], Nogales V, et al. Oncotarget. 2016 [25] and more). Hence, factors that regulate SLFN11 expression are legitimate therapeutic targets for further testing (Murai J, et al. Pharmacol Ther. 2019 [4] and Tang SW, et al. Clin Cancer Res. 2015 [52]). Therefore, at the end of the discussion of our revised manuscript, we would like to retain this point to foster future studies by independent investigators.

6. Figure 4. B the quality of Western blot result is poor. The bands of Actin and H3K9ac in FL318 cells are vague. RT-PCR results need to be statistically analyzed.

(Answer) Thank you for carefully examining our results. As suggested, we performed new Western blots and used better images in the revised manuscript (Fig 4C below). Regarding RT-PCR, we performed t-test for all the genes in the heat map (Fig 4B below) and added asterisks to represent the significant changes. We also omitted the bar-graphs for fold change of SLFN11 in Figure 4B right of the original manuscript to avoid showing redundant data.

(Figure 4C)

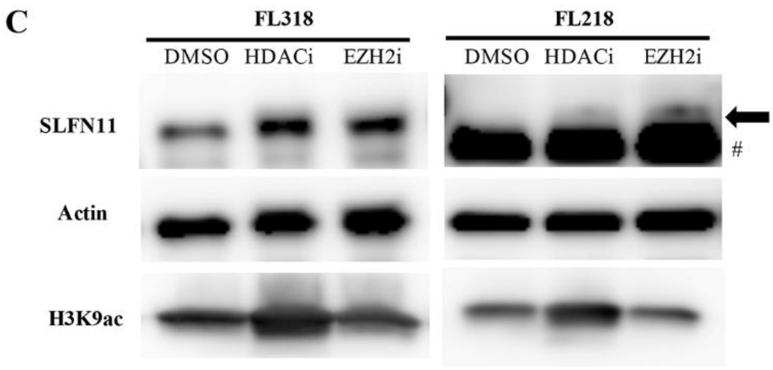

(Figure 4B)

B

Drug	Cell Line	*SLFN11*	ABC associated genes *PTPN1*	*ENTPD1*	*IRF4*	*ETV6*	GCB associated genes *MYBL1*	*ITPKB*	*BCL6*	*LRMP*
EZH2i	SU-DHL6	2.91	1.52*	3.08*	2.20	3.43*	0.600*	0.974	1.40	1.29*
	Daudi	7.70	1.16*	22.7	3.90	1.46	0.475*	1.05	0.765	0.546*
	FL18	1.20	0.948	1.01	1.13	1.68	0.927	0.749*	0.846	0.776*
	FL218	4.47	1.94*	2.35	6.84	11.4*	1.13	1.19	1.12	1.49
	FL318	1.06	1.73	33.5*	1.61	0.960	0.437*	0.556*	0.228*	0.504*
HDACi	SU-DHL6	17.8*	1.66*	2.99*	1.76*	3.11*	1.46*	1.36*	0.601	1.08
	Daudi	14.7*	1.63*	6.09	1.08	1.54	0.625*	0.719*	0.850	0.797*
	FL18	2.09*	1.57*	1.58*	2.46*	2.27*	1.59*	1.19	0.784*	0.914
	FL218	5.09	1.48*	2.74*	2.11	4.84	0.836*	0.548*	0.774*	0.639*
	FL318	2.63*	1.65*	7.64*	2.08*	1.55*	2.68*	1.06	1.35*	2.52*

Legend: x5< ; x2< ; x1< ; <x1 ; <x0.5 ; <x0.2

Reviewer #2: *In this manuscript, the authors gave us a general investigation that SLFN11 was downregulated in germinal center B cells, and epigenetic modifiers EZH2 inhibitor and HDAC inhibitor could elevate its expression, which drove the cells more sensitive to cytosine arabinoside treatment.*

Comments:

1. in the text, some of the conclusions were over interpreted, like line 182-183, line 262.

(Answer) Thank you very much for these suggestions. Regarding the conclusions in lines 182-183, we revised the sentence to "Thus, among *SLFN*s, *SLFN11* uniquely showed parallel expression profile compared to *PRDM1* and *XBP1* and reverse expression profile with respect to *PAX5* across B-cell development." For line 262, to avoid overinterpretation we revised the sentence to "These results consolidate the finding of differential expression of *SLFN11* between ABC-DLBCL and GCB-DLBCL in clinical samples."

2. No any evidence to show the mechanism of SLFN11 downregulation;

(Answer) Thank you for raising an important point. To determine whether epigenetics is a mechanism for SLFN11 downregulation, we examined the correlation between *SLFN11* expression and DNA methylation level of the *SLFN11* promoter in B-cell-derived malignancies. Overall, we observed a significant reverse correlation between *SLFN11* expression and DNA methylation across B-cell derived cancers (revised Figure 4A below). Interestingly, *SLFN11* expression of GCB-DLBCL lines (▲) is suppressed without promoter DNA methylation, implying that histone modifications rather than DNA methylation are plausible suppressors of *SLFN11* suppression in GCBs. Consistent with this possibility, we found that HDAC inhibitor or EZH2 inhibitor reactivate *SLFN11* expression in GCB cells (revised Figure 4B-C).

(Figure 4A)

A

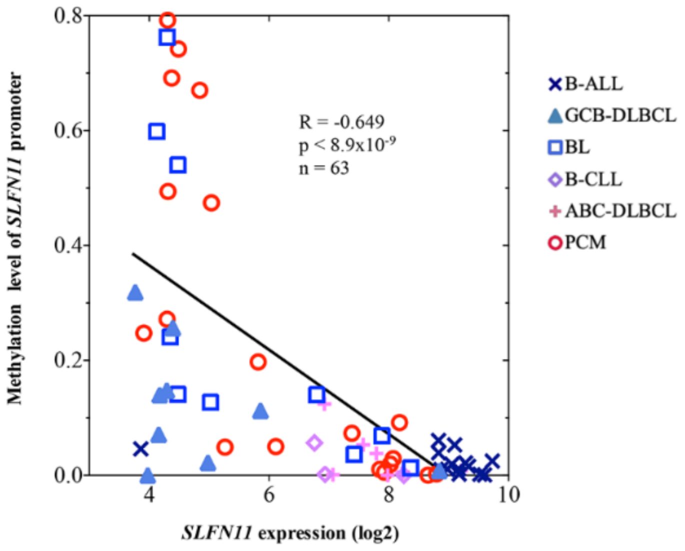

3. Knockdown or overexpression of SLFN11 gene in ABC or GCB cell lines to study the sensitivity of these cells to arabinoside treatment.

(Answer)

Thank you for your suggestions. To answer your question, we worked hard to make SLFN11 KO cells in SU-DHL6 and other GCB cell lines. However, SU-DHL6 did not express SLFN11 high enough to be detected by WB under the treatments of epigenetic modifiers, and we were unable to validate candidate clones of SLFN11-KO. For follicular lymphoma cell lines, FL18, FL218 and FL318 did not form single colonies after the transfection of SLFN11-KO vectors. For these reasons, we have been unable to establish SLFN11-KO cell lines in the GCB cells used in this study. Alternatively, to relate *SLFN11* expression with the activity of AraC in GCB cells, we mined publicly available data on *SLFN11* expression and drug activity of AraC. *SLFN11* expression was found significantly correlated to the activity of AraC in B-cell-derived cancer cell lines. These data have been included in the revised Figure 5A (below). This result is consistent with a previous report showing that the synergistic combination of HDACi with the replication selective DNA damaging agents (camptothecin) is SLFN11-dependent (i.e., the synergy was not observed

in SLFN11-KO cells) (Tang SW, et al. Clin Cancer Res. 2018. [26]). Based on these observations, we consider that activation of SLFN11 expression by epigenetic modifiers can enhance the activity of AraC. Nevertheless, since we failed to generate KO or OE cells in GCB cells, we rephrased our conclusion in the revised manuscript and stated, "These results indicate that the combination of the epigenetic modifiers that enhance SLFN11 expression can improve the response in GCB lymphoma cells to AraC."

(Figure 5A)

A

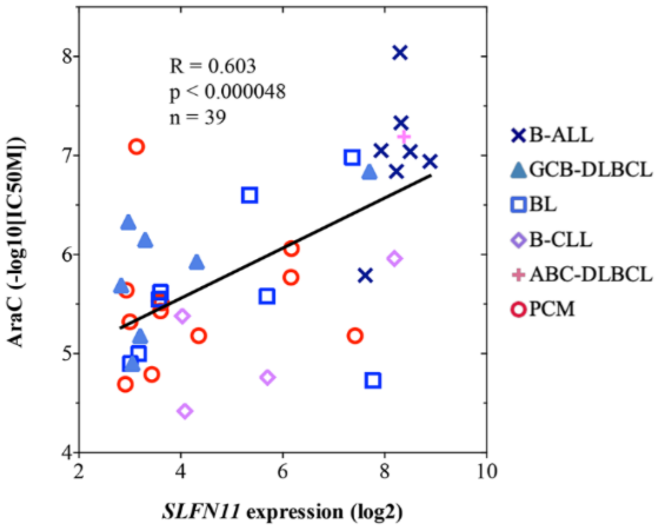

Supplement: Attachment — Submitted filename: Response to Reviewers.pdf [file pone.0237554.s008.pdf]
