# Supplementary material for: Epigenetic suppression of SLFN11 in germinal center B-cells during B-cell development
Source: PLoS One. 2021 Jan 29;16(1):e0237554. doi: 10.1371/journal.pone.0237554 (PMC7846023; doi:10.1371/journal.pone.0237554)

Point-by-point answers to the reviewers

The list of changes and answers are indicated below (in blue) with the comments of the reviewers included in *italic* before our answers to facilitate the review process.

Reviewers' comments to the Author

Reviewer #1: *SLFN11 plays key role in execute cancer cells harboring replicative stress induced by DNA damaging agents while the roles of SLFN11 under physiological conditions are not widely studied. B-cells undergo gene editing at variable regions of the immunoglobulin gene loci during the development and maturation. During this process, B-cells are physiologically exposed to genotoxic stress caused by somatic hypermutations and class-switch recombination. Such genotoxic tress is introduced particularly to centroblasts and centrocytes in germinal centers (GCs) of lymph nodes. Thus, Germinal center B-cells (GCBs) undergo somatic hypermutations and class-switch recombination, which can cause physiological genotoxic stress. Hence, Fumiya Moribe etal tested whether the expression of SLFN11 is needed to be controlled during B-cell development to avoid SLFN11- dependent cell death in cells undergoing genomic rearrangements. They performed several mRNA and Protein expression analysis on SLFN11 by using cell lines of different stages of normal B cells and various types of B-cell lymphoma or normal human lymphatic tissues and some Cell viability experiments and found the following results: SLFN11 mRNA level was low in both normal GCBs and GCB-DLBCL (GCB like-diffuse large 4 B-cell lymphoma). Low SLFN11 expression in GCBs and high SLFN11 expression in plasmablasts and plasmacytes. The EZH2 and HDAC epigenetic modifiers upregulated SLFN11 expression in GCB-derived lymphomas and made them more susceptible to cytosine arabinoside. Overall the experimental designs are logical and some data mincing are meaningful. The major concern is that the article is focus on the expression and function of SLFN11 during B cell development, especially in the GCB-derived lymphomas and their susceptibility to cytosine arabinoside. Therefore, SLFN11 knock down or overexpression in some of these cells are necessary for further studying. A few minor comments are listed below:*

1. *Figure 4C, the western blot bands are not clear. Especially H3K9AC. The right SLFN11 band should be divided with the non specific bands by extending the running time of SDS page gels.*

(Answer) Thank you for carefully examining our results. As suggested, we repeated the western blot for FL218 for multiple times. However, due to the very low expression of SLFN11 in FL218, we were unable to divide the SLFN11 band from the non-specific band. Thus, we tried to test SLFN11 protein level in another cell line. We could observe SLFN11 bands in FL18 and its upregulation by HDACi, which is consistent with the qPCR results. Hence, we included the western blot results of FL18 and excluded those of FL218 in the updated manuscript. As for the blot of H3K9Ac, we confirmed that HDACi but not EZH2 inhibitor increased the acetylation level of H3K9, which is reasonable since HDACi targets broad acetylation sites of histones while EZH2 inhibitor targets exclusively H3K27. Hence, we keep the previous blot of H3K9Ac in FL318 and added a comparable result of FL18.

(Figure 4C)

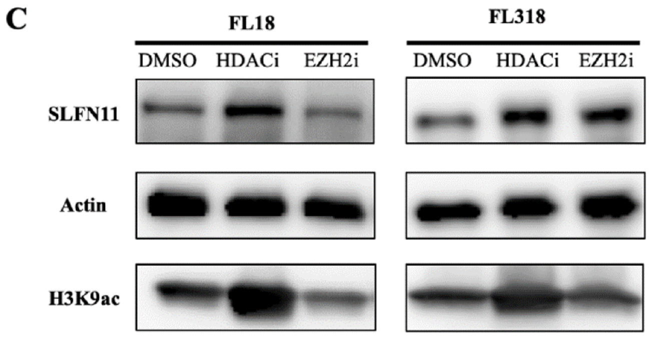

2. *Figure 5, SLFN11 should be knockdown by shRNA (SiRNA) or overexpressed in ABC or GCB cell lines to study the sensitivity of these cells to arabinoside treatment.*

(Answer) Thank you very much for your suggestions. We generated SLFN11-overexpressing SU-DHL6 cell line by using piggy back tetON system (SU-DHL6 tetON SLFN11 cells). In this system, we were able to induce SLFN11 expression when treating the cells with doxycycline. SLFN11 induction in SU-DHL6 tetON SLFN11 cells was confirmed by western blot and immunofluorescence (Fig5D). Using this cell line, we examined whether SLFN11 sensitizes the cells to DNA damaging agents. We treated SU-DHL6 tetON SLFN11 cells with arabinoside (AraC) and observed that SU-DHL6 tetON SLFN11 cells were more sensitive to AraC (Fig 5E left).

We also found *SLFN11* expression was correlated to the activity of camptothecin (CPT) in B-cell cancer cell lines in the GDSC database (Fig5A right). SLFN11 overexpression sensitized the cells to CPT as well (Fig5E right). To consolidate the role of SLFN11 in cellular sensitivity to DNA damaging agents in GCBs, we included these data in the revised manuscript.

(Figure 5D)

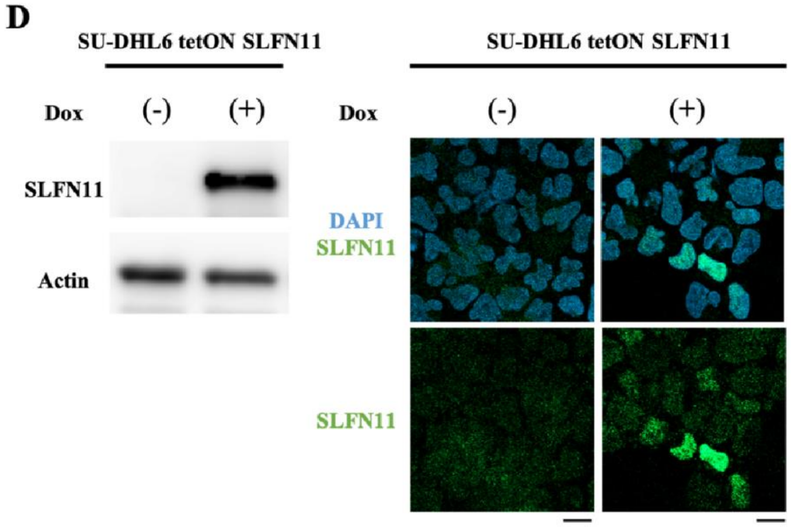

(Figure 5E)

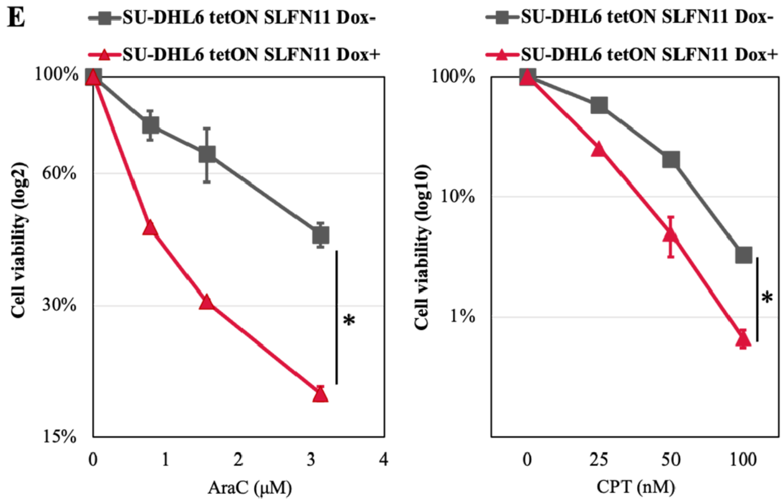

(Figure 5A)

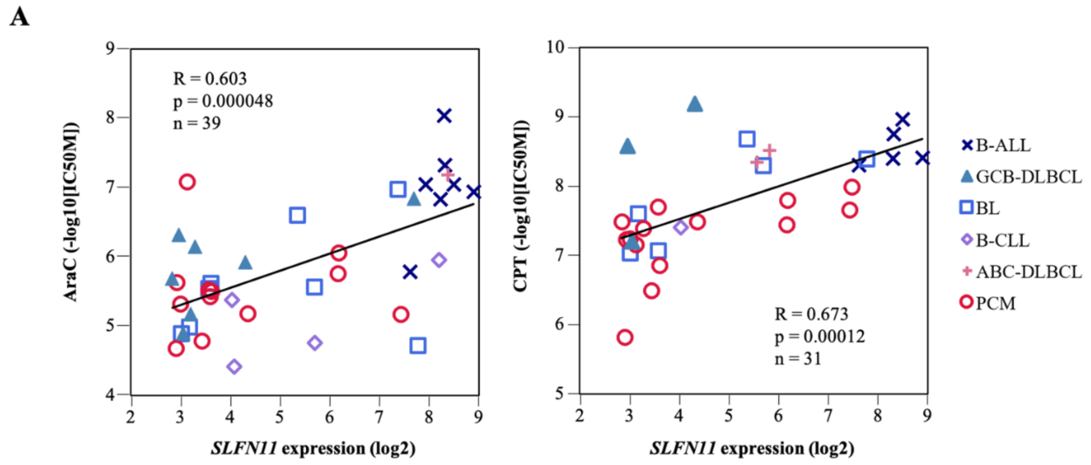

Supplement: Attachment — Submitted filename: Response to Reviewers.pdf [file pone.0237554.s009.pdf]
